# Clinical and histopathological study of renal biopsy in Nepalese children: A single center experience

**DOI:** 10.1371/journal.pone.0276172

**Published:** 2022-10-27

**Authors:** Manim Amatya, Anil Dev Pant

**Affiliations:** Pathology Department, Grande International Hospital, Kathmandu, Nepal; University of Bari: Universita degli Studi di Bari Aldo Moro, ITALY

## Abstract

**Background:**

Glomerular diseases are important causes of morbidity and mortality among children in developing countries. Renal biopsy is the gold standard for determining histological diagnosis, prognosis, and therapy options. This study aimed to investigate the clinical, histopathological, and direct immunofluorescence findings of renal biopsy results in Nepalese children under 18 years old.

**Methods:**

In this retrospective cross-sectional study, the available data from children who had undergone kidney biopsy between 2016 and the end of 2020 were evaluated. Demographic data, indications of biopsy, histopathologic findings, and direct immunofluorescence findings were collected and presented.

**Results:**

The mean age of the patients was 12.14 ± 4.07 years. Male/female ratio was 1:1. The majority of biopsy cases were aged between 11–15 years of age. The most common indication for biopsy in children was nephrotic syndrome (68.25%). Lupus nephritis with 28 cases (22.22%) had the highest frequency in overall renal biopsies. Minimal change disease (MCD) with 22 cases (17.46%) followed by Ig A nephropathy with 16 cases (12.69%) were the most frequent primary glomerulonephritis. Lupus nephritis showed full house positivity, and MCD showed full house negativity in all Direct immunofluorescence (DIF) parameters, whereas immunoglobulin A nephropathy showed 100% positivity in Ig A in DIF.

**Conclusions:**

Nephrotic syndrome was the most common indication for renal biopsy. The most common primary glomerulonephritis was MCD, while secondary glomerulonephritis was lupus nephritis. Clinical data, light microscopy, and direct DIF played an integral role in the overall final diagnosis.

## Introduction

Glomerular diseases are significant causes of morbidity and mortality and a considerable burden on health services in developing countries. Diagnosis and classification of glomerular disease pose a significant challenge to the pathologist due to the complexity and variety of these lesions [1].

Many children with kidney disease do not need a kidney biopsy for diagnosis. However, renal biopsy remains an essential tool for establishing a histological diagnosis, determining disease severity and prognosis, planning, and monitoring treatment modalities in some patients. Nephrotic syndrome with steroid resistance, steroid dependence or atypical features, recurrent hematuria, systemic lupus erythematous nephritis (SLE), Henoch Schonlein purpura nephritis (HSP), and unexplained acute kidney injury (AKI) are few indications of renal biopsy in children [2].

The knowledge of epidemiology, a pattern of renal disease, and histopathological association with clinical features in particular regions are different worldwide due to differences in race, socio-economical gaps, and guidelines for performing kidney biopsies in children [3].

There is a scarcity of data regarding the prevalence of glomerular diseases in children and their association with histopathological findings in Nepal. Therefore, this study was conducted to describe the spectrum of glomerular diseases in pediatric age groups, indications for renal biopsy, their histopathological appearance, and their clinical correlation based on light microscopic and immunofluorescence findings.

## Materials and methods

### Patients

This was a retrospective study of renal biopsy samples received in the Pathology Department of Grande International Hospital of children aged less than 18 years who underwent percutaneous renal biopsies between January 2016 and December 2020. All biopsies were performed by nephrologists under ultrasound guidance. Two tissue cores were collected, one for light microscopy, which was kept in 10% neutral buffered formalin, and another core for immunofluorescence, which was held in normal saline and then transported to our laboratory. Renal biopsies were examined using light microscopy and DIF.

### Indications for renal biopsy

The indication for kidney biopsy was categorized into seven clinical categories: nephrotic syndrome, nephritic syndrome, non-nephrotic proteinuria, isolated hematuria, nephritic proteinuria + hematuria, renal failure, and miscellaneous (SLE, HSP, HIV, and Goodpasture Disease).

### Ethical consideration

The study was approved by the Institutional Review Committee of Grande International Hospital and carried out in accordance with the Declaration of Helsinki. The need for informed consent was waived by “The Institutional Review Committee of Grande International Hospital (IRC-GIH)” IRB due to the retrospective nature of the study.

### Data collection

Medical records of all children who underwent renal biopsy were retrieved from the medical record database and analyzed. The following data for each patient: name, age, sex, indication of kidney biopsy, histopathological findings, histopathological diagnosis, and laboratory investigations such as serum creatinine, 24-hour urinary protein, urine microscopy, anti-double-stranded DNA antibody, antinuclear antibody (ANA), complements levels 3 and 4 (C3, C4) were noted.

### Histopathological analysis

For the light microscopic study, tissue processing was performed from formalin-fixed and paraffin-embedded (FFPE) tissues. Tissue sections were cut at 4μm thickness and staining was done using hematoxylin and eosin (HE), periodic acid- Schiff (PAS), Congo red stain, and Masson’s Trichrome stain. For DIF, Dako reagent with appropriate dilution was prepared and a fresh frozen tissue specimen was cut in a cryostat. Air dry slides were stained for 20 minutes. It was prewashed in Phosphate buffered saline (PBS) for 5–10 minutes. 50–100 μl of working dilution of the Fluorescein isothiocyanate (FITC) conjugated antibody was applied. The slide was incubated at room temperature for 30 minutes in a moist chamber and was washed in Phosphate buffered saline (PBS) for 3 changes, 5 minutes each. Cover slip was used with glycerol-based mounting media. Immunoglobulins and complement analyses were done using an immunohistochemical antibody panel to immunoglobulins G, M, A (IgG, IgM, IgA), C3, complement 1 q (C1q), and also to kappa and lambda light chains. All kidney biopsy specimens obtained were prepared as per the standard protocol and examined by renal pathologists using Leica DM 1000 LED microscope. Analysis included light microscopy (LM) and immunofluorescence (IF). However, electron microscopy (EM) was not systematically performed, as this facility was not available in our institution.

### Statistical analysis

Data analysis was done with SPSS 17.0 and percentages and proportions of indications, demographic parameters, histopathological findings, histopathological diagnosis, and their clinical correlation were calculated.

## Results

### Demographic data and clinical presentation

**Fig 1:**
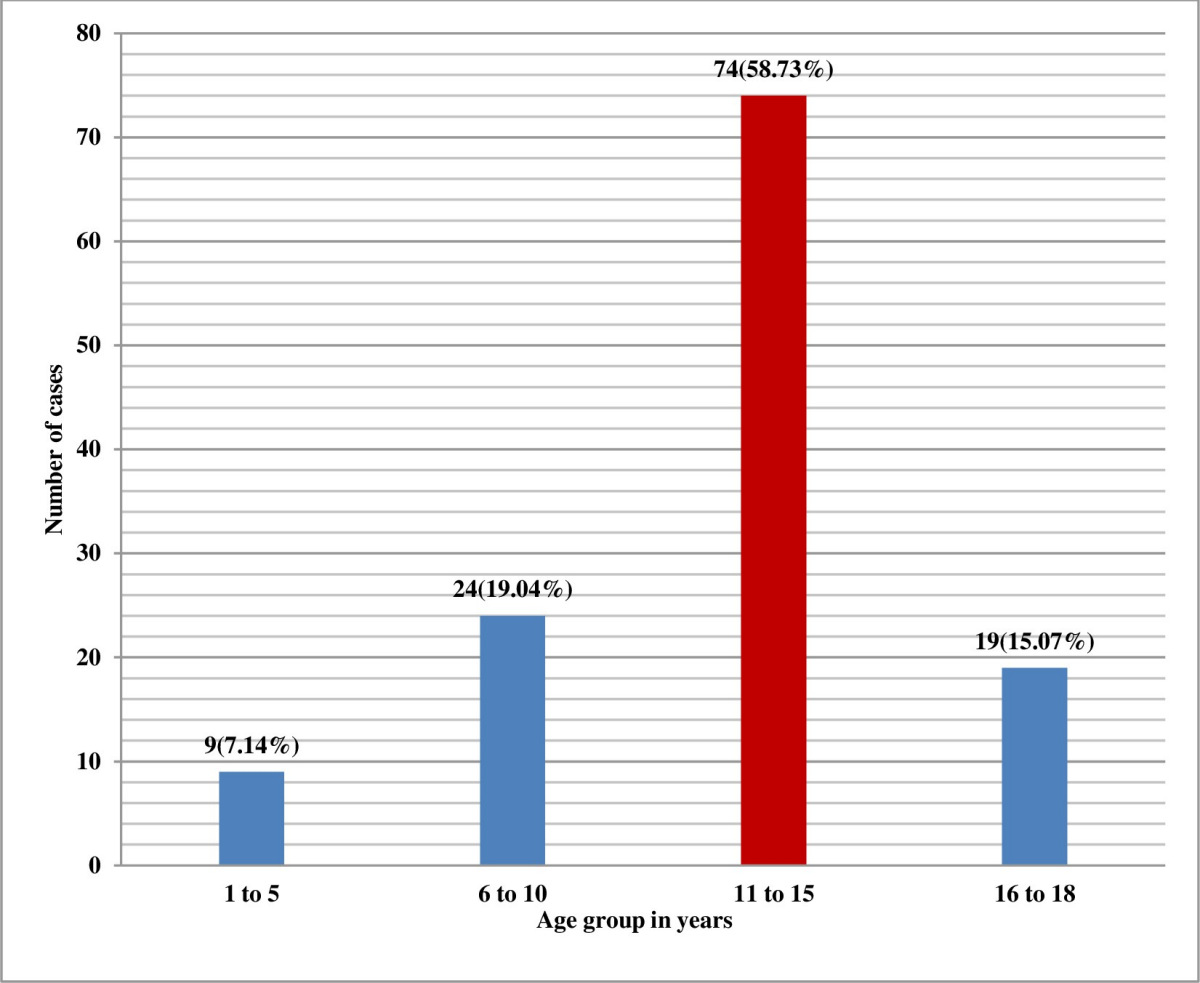
Age distribution of paediatric kidney patients (N = 126).

A total of 126 patients were biopsied during the study period of five years. Of the 126 cases, 63 (50%) were boys and 63 (50%) were girls. The mean age was 12.14 ± 4.07 years and the age ranged from 1 year to 18 years as shown in Fig 1. Females had a mean age of 12.90 ± 3.81 years and males 11.38 ± 4.21years.

**Fig 2:**
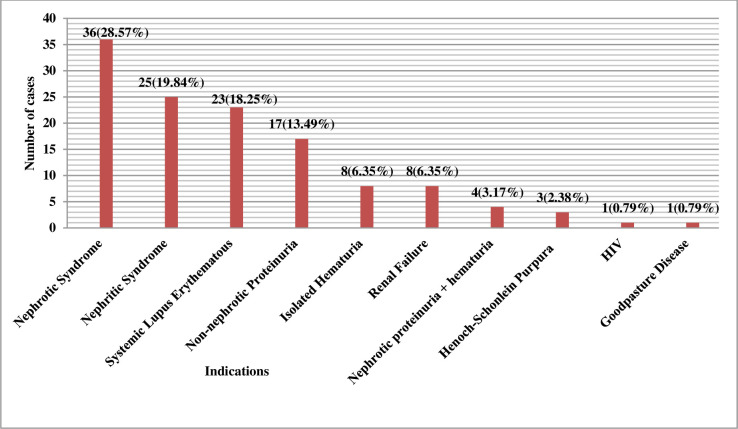
Clinical indications of renal biopsy in children (N = 126).

The most common clinical indications for renal biopsy were nephrotic syndrome 36 (28.57%), followed by nephritic syndrome 25 (19.8%) and Systemic Lupus Erythematosus (SLE) 23 (18.25%), respectively. All these cases of SLE were positive for ANA and dsDNA. The frequency of causes of renal biopsies in the studied children is illustrated in Fig 2.

Of 36 patients with nephrotic syndrome, 24 (68.25%) were steroid-resistant, 11 (30.55%) were steroid-sensitive, and 1 (2.78%) was frequently relapsing.

ANA/ds-DNA was done in 54 cases, of which 23 (42.59%) cases were positive.

### Histopathological diagnoses

The results showed that primary glomerular disease was seen in 86 cases (68.25%) of all 126 biopsied patients. It was followed by secondary glomerular disease and hereditary glomerular disease which were seen in 37 cases (29.36%) and three cases (2.39%), respectively. The histopathological findings in kidney biopsied patients are shown in Table 1.

**Table 1:** Histopathological diagnosis (N = 126).

FINAL DIAGNOSES	FREQUENCY N (%)
**Primary Glomerulonephritis**	**86(68.25%)**
**Minimal Change Disease**	22(17.46%)
**IgA Nephropathy**	16(12.69%)
**Focal Segmental Glomerulosclerosis**	13(10.31%)
**Crescentic Glomerulonephritis**	10(7.94%)
**Type I (Anti-GBM)**	3(30%)
**Type II (Immune complex)**	2(20%)
**Type III (Pauci immune)**	5(50%)
**Membranoproliferative Glomerulonephritis**	10(7.94%)
**Post-infectious Glomerulonephritis**	10(7.94%)
**Membranous Nephropathy**	3(2.38%)
**IgM Nephropathy**	2(1.58%)
Secondary Glomerulonephritis	**37(29.36%)**
**Lupus Nephritis**	28(22.22%)
**Class IV (Diffuse proliferative)**	16(72.72%)
**Class III (Focal proliferative)**	11(39.28%)
**Class V (Membranous)**	1(3.57%)
**Thrombotic Microangiopathy**	3(2.38%)
**Henoch-Schonlein Purpura Nephritis**	2(1.59%)
**Acute Kidney Injury**	2(1.59%)
**Tubulointerstitial Nephritis**	1(0.79%)
**HIV-Associated Immune Complex Kidney Disease**	1(0.79%)
**Hereditary**	**3(2.39%)**
**Alport Syndrome**	2(1.59%)
**Thin basement Membrane Disease**	1(0.79%)
**Total**	**126(100%)**

As seen in the table, SLE with 28 cases (22.22%) was the most common disease. It was followed by MCD, IgA nephropathy, FSGS, membranoproliferative glomerulonephritis, postinfectious glomerulonephritis, and crescentic glomerulonephritis which were seen in 22 (17.46%), 16 (12.69%), 13 (10.31%), 10 (7.94%), 10 (7.94%), and 10 (7.94%) samples respectively. Among cases of hereditary nephritis, all had a family history. Two cases had clinical and histopathological findings suggestive of Alport’s and one case had only mild hematuria, possibly Thin basement membrane disease.

Most of the patients 73(57.93%) were in the age group 11 to 15 years. Age-wise breakdown of histopathological diagnoses are shown in Table 2.

**Table 2:** Age wise histopathological diagnoses (N = 126).

FINAL DIAGNOSES	1–5 years	6–10 years	11–15 years	16–18 years	Total N(%)
**Lupus Nephritis**		5(17.86%)	19(67.86%)	4(14.28%)	**28(22.22%)**
**Minimal Change Disease**	5(22.73%)	3(13.63%)	10(45.45%)	4(18.18%)	**22(17.46%)**
**IgA Nephropathy**	1(6.25%)	2(12.5%)	9(56.25%)	4(25%)	**16(12.69%)**
**Focal Segmental Glomerulosclerosis**	1(7.69%)	4(30.77%)	7(53.85%)	1(7.69%)	**13(10.32%)**
**Crescentic GN**		2(20%)	8(80%)		**10(7.94%)**
**Membranoproliferative GN**			10(100%)		**10(7.94%)**
**Post-infectious GN**		4(40%)	6(60%)		**10(7.94%)**
**Membranous Nephropathy**				3(100%)	**3(2.38%)**
**Thrombotic Microangiopathy**		2(66.67%)		1(33.33%)	**3(2.38%)**
**Acute Kidney Injury**		1(50%)		1(50%)	**2(1.59%)**
**Henoch-Schonlein Purpura Nephritis**			2(100%)		**2(1.59%)**
**IgM Nephropathy**	1(50%)			1(50%)	**2(1.59%)**
**Alport Syndrome**		1(50%)		1(50%)	**2(1.59%)**
**HIV-Associated Immune Complex Kidney Disease**			1(100%)		**1(0.79%)**
**Thin basement Membrane Disease**	1(100%)				**1(0.79%)**
**Tubulointerstitial Nephritis**			1		**1(0.79%)**
**Total**	**9(7.14%)**	**24(19.05%)**	**73(57.93%)**	**20(15.87%)**	**126(100%)**

### Histopathological findings

#### Glomeruli

**Fig 3:**
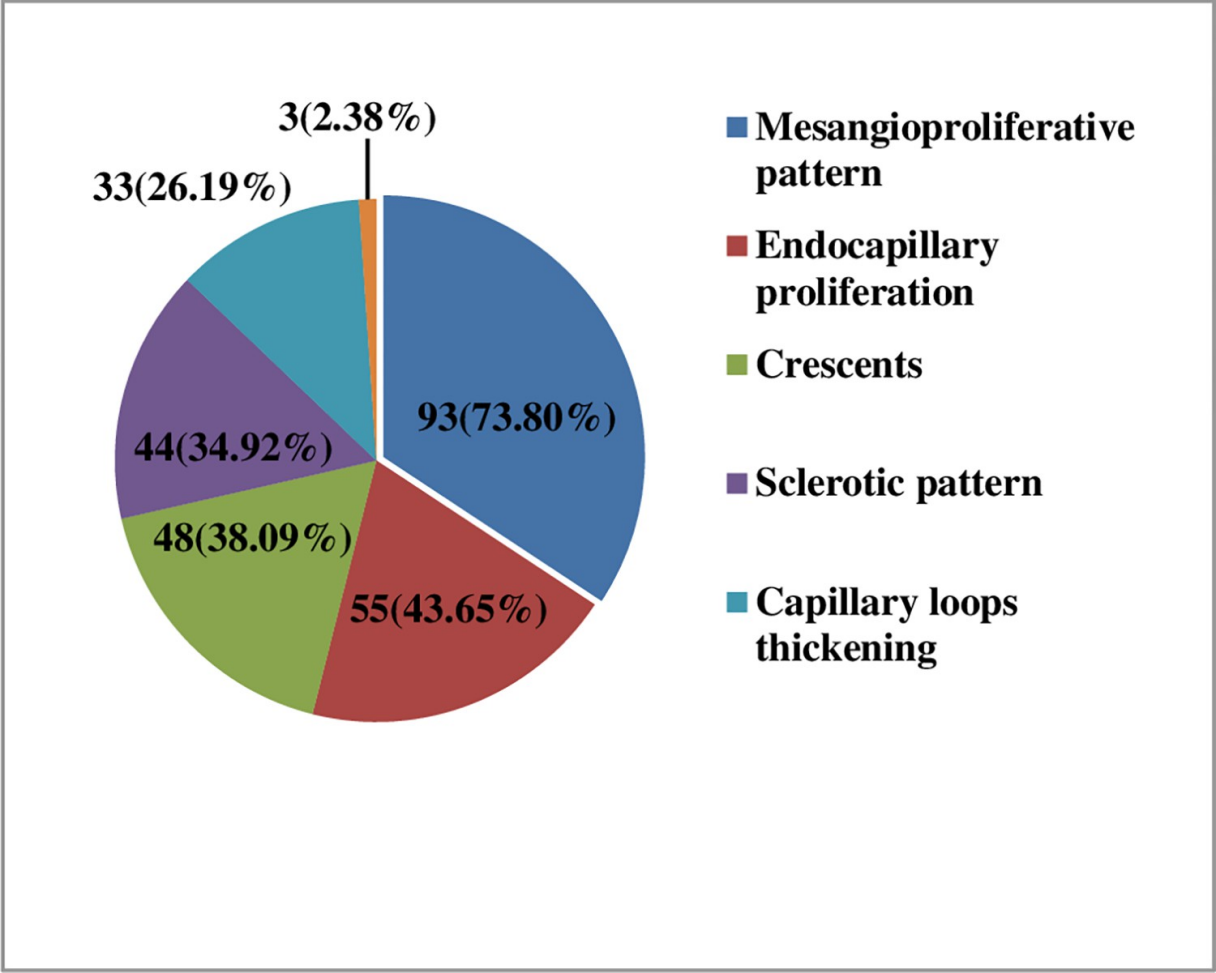
Histopathological findings in glomeruli.

Mesangioproliferative pattern was observed in 93 (73.8%) cases, endocapillary proliferation in 55 (43.65%) cases, crescents in 48 (38.09%) cases, sclerotic pattern in 44 (34.92%) cases, capillary loop thickening in 33 (24.19%) cases and thrombi in capillaries with fibrinoid necrosis seen in 3 (2.38%) cases. (Fig 3).

#### Interstitium

Unremarkable/Mild tubular atrophy with fibrosis was seen in 115 (91.26%) cases, moderate tubular atrophies with fibrosis were seen in 10 (7.93%) cases, and severe tubular atrophy with fibrosis was in 1 (0.79%) cases. RBC casts were seen in 11 (8.73%) cases, hyaline casts were seen in 6 (4.76%) cases, and focal calcification was seen in 1 (0.79%) case.

#### Tubules

There was acute mild injury in renal tubules in 89 (70.63%) cases, acute moderate injury in 29 (23.01%) cases, acute severe injury in 3 (2.38%) cases, and 5 (3.96%) cases were unremarkable.

#### Arteries

Intimal thickening was observed in 14 (11.11%) cases, hyalinosis in 9 (7.14%) cases, endothelial swelling in 1 (0.79%) cases, and fibrinoid necrosis in 1 (0.79%) case. 108 (85.71%) cases did not show significant vascular lesions.

#### Histopathological lesions in lupus nephritis

According to the International Society of Nephrology and the Renal Pathology Society (ISN/RPS) 2016 classification of Lupus nephritis, out of total 28 cases of Lupus nephritis, the maximum cases were categorized as Class IV [16 (57.1%)], followed by Class III [10 (35.7%)], Class II [1 (3.6%)], Class V [1 (3.6%)].

**Fig 4:**
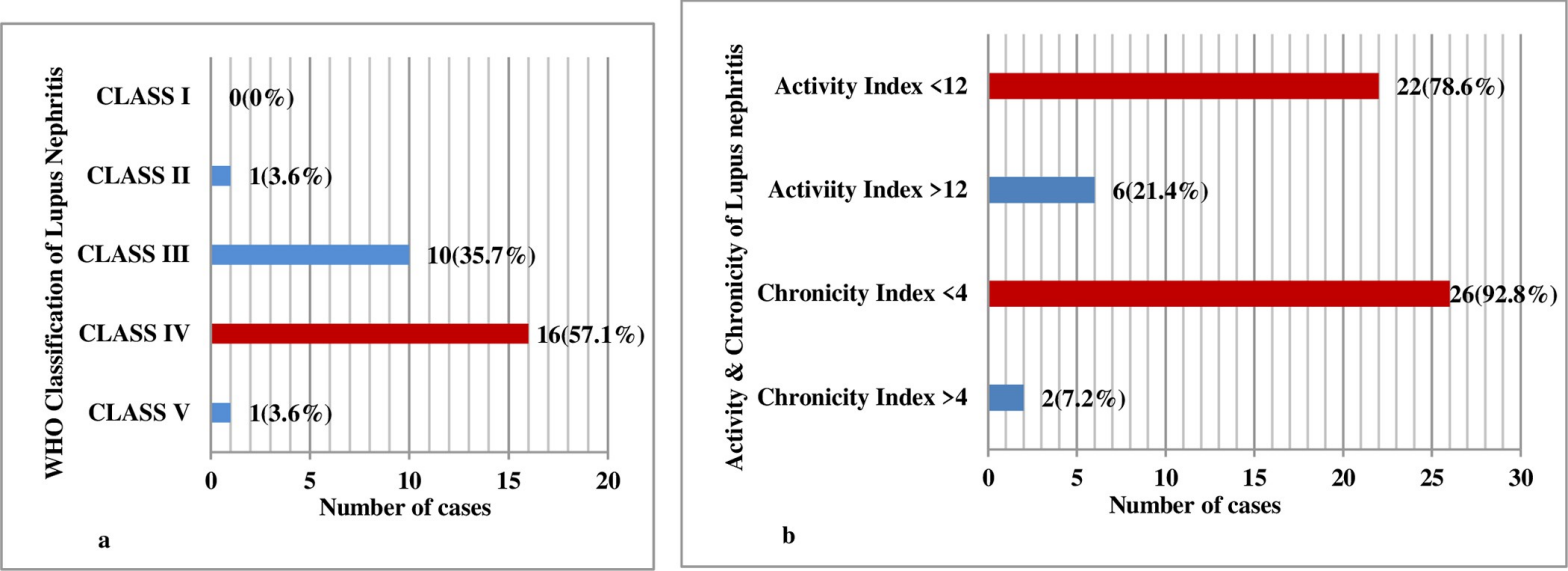
a WHO classification of Lupus Nepritis (N = 28). b. Activity chronicity index of Lupus Nephritis (N = 28).

According to the modified NIH activity chronicity index in lupus nephritis, Activity index <12 was in 22 (78.6%) cases and activity index >12 was seen in 6 (21.4%) cases. Similarly, Chronicity Index <4 was seen in 26 (92.8%) cases, and the chronicity Index >4 was seen in 2 (7.2%) cases. These findings along with the activity chronicity index of lupus nephritis are illustrated in Fig 4A and 4B.

#### Classification of crescentic glomerulonephritis

**Fig 5:**
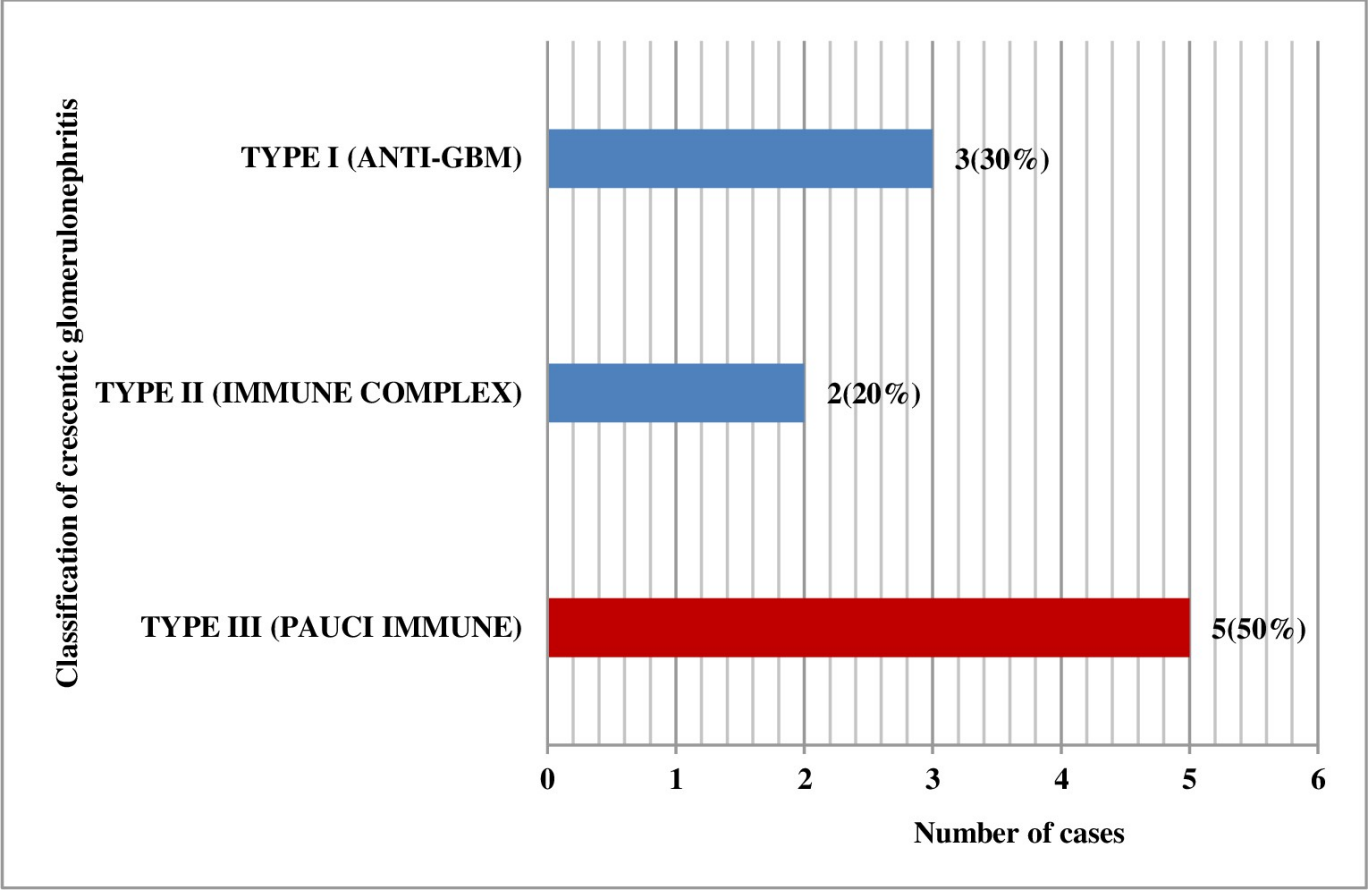
Classification of crescentic glomerulonephritis (N = 10).

Out of 10 cases of crescentic glomerulonephritis, the highest frequencies were of Type III (Pauci immune) 5 (50%) followed by Type I (Anti-GBM) 3 (30%) and Type II (Immune complex) 2 (20%) as shown in Fig 5.

#### Histopathological findings in IgA nephropathy

**Fig 6:**
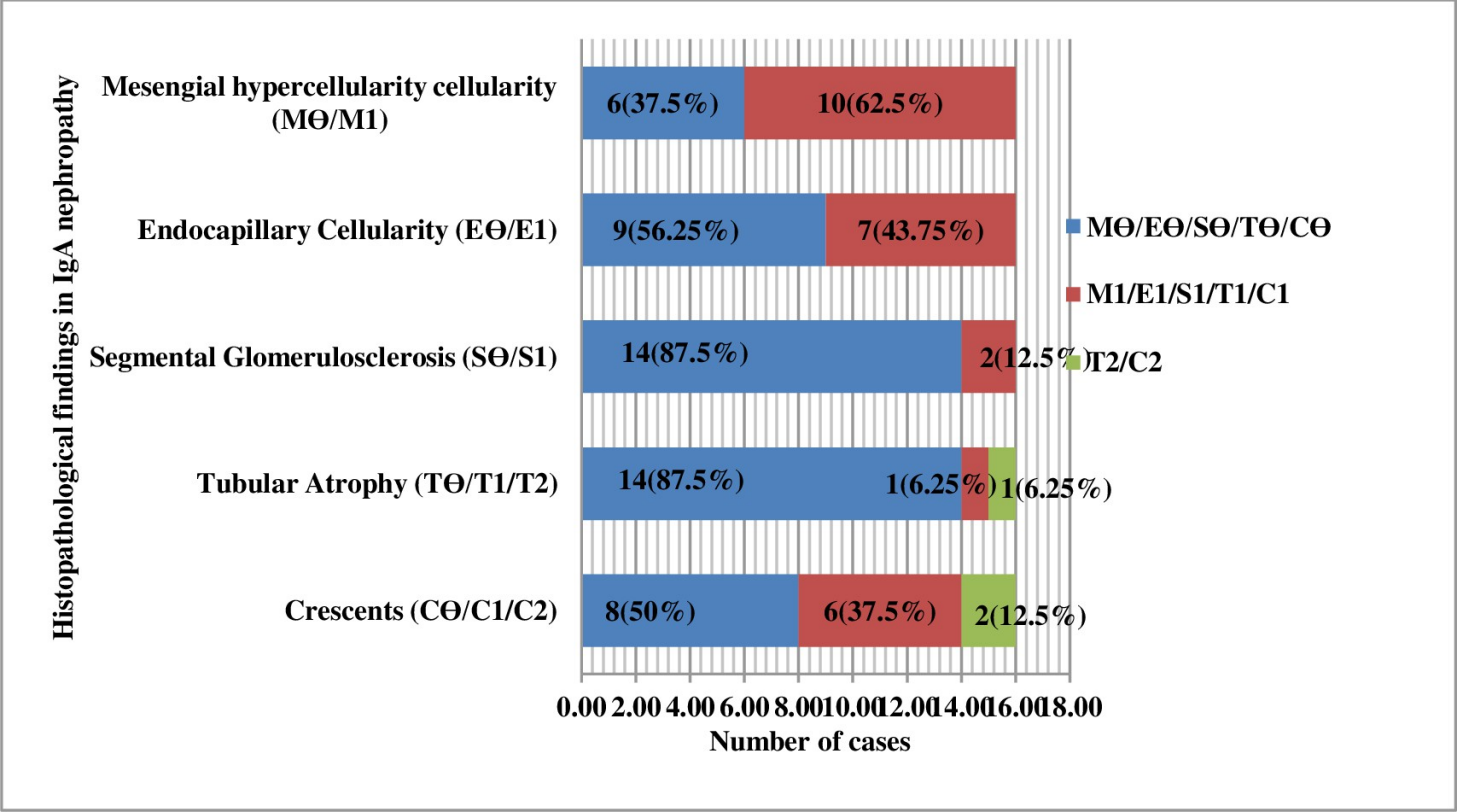
Histopathological findings of Ig A nephropathy (N = 16).

According to the updated Oxford Classification (MEST-C) 2016, mesangial hypercellularity, MӨ (<50% of glomeruli showing mesangial hypercellularity) was seen in 6(37.5%) cases and M1 (>50% of glomeruli showing mesangial hypercellularity) was seen in 10 (62.5%) cases. EӨ (no endocapillary hypercellularity) was seen in 9 (56.25%) cases and E1 (any glomeruli showing endocapillary hypercellularity) in 7 (43.75%) cases. SӨ (Segmental glomerulosclerosis absent) were in 14 (87.5%) cases and S1 (glomerulosclerosis present in any glomeruli) in 2 (12.5%) cases. TӨ (Tubular atrophy or interstitial fibrosis in 0–25% cortical area) was seen in 14 (87.5%) cases, T1 (Tubular atrophy or interstitial fibrosis in 26–50% cortical area) was seen in 1 (6.25%) case and T2 (Tubular atrophy or interstitial fibrosis in >50% cortical area) was seen in 1 (6.25%) case. CӨ (cellular or fibro cellular crescents absent) was seen in 8 (50%) cases, C1 (crescents in 0–25% of glomeruli) in 6 (37.5%) cases and C2 (crescents in >25% of glomeruli) in 2 (12.5%) cases. (Fig 6).

The pattern of Glomerular disease according to clinical indications is shown in Table 3.

**Table 3:** Spectrum of glomerular diseases and their clinical presentation (N = 126).

INDICATIONS FINAL DIAGNOSES	Frequency	Nephrotic Syndrome	Nephritic Syndrome	SLE	Non-nephrotic Proteinuria	Isolated Hematuria	Renal Failure	Nephrotic proteinuria + hematuria	HSP	HIV	Goodpasture Disease
**Lupus Nephritis**	**28(22.22%)**	1(2.77%)	1(4%)	22(95.65%)	2(11.76%)	1(12.5%)	1(12.5%)				
**Minimal Change Disease**	**22(17.46%)**	19(52.77%)	2(8%)		1(5.88%)						
**IgA Nephropathy**	**16(12.69%)**	3(8.33%)	3(12%)		3(17.64%)	3(37.5%)	2(25%)	1(25%)	1(33.33%)		
**Focal Segmental Glomerulosclerosis**	**13(10.32%)**	8(22.22%)	2(8%)		1(5.88%)	1(12.5%)		1(25%)			
**Crescentic GN**	**10(7.94%)**	1(2.77%)	1(4%)		3(17.64%)	1(12.5%)	2(25%)	1(25%)			1(100%)
**Membranoproliferative GN**	**10(7.94%)**	2(5.55%)	4(16%)		2(11.76%)		1(12.5%)	1(25%)			
**Postinfectious GN**	**10(7.94%)**		8(32%)		2(11.76%)						
**Membranous Nephropathy**	**3(2.38%)**	1(2.77%)		1(4.34%)	1(5.88%)						
**Thrombotic Microangiopathy**	**3(2.38%)**				1(5.88%)		2(25%)				
**Acute Kidney Injury**	**2(1.59%)**		1(4%)		1(5.88%)						
**Henoch-Schonlein Purpura Nephritis**	**2(1.59%)**								2(66.66%)		
**IgM Nephropathy**	**2(1.59%)**		1(4%)			1(12.5%)					
**Alport Syndrome**	**2(1.59%)**	1(2.77%)	1(4%)								
**HIV-Associated Immune Complex Kidney Disease**	**1(0.79%)**									1(100%)	
**Thin Basement Membrane Disease**	**1(0.79%)**					1(12.5%)					
**Tubulointerstitial Nephritis**	**1(0.79%)**		1(4%)								
**Total**	**126(100%)**	36(100%)	25(100%)	23(100%)	17(100%)	8(100%)	8(100%)	4(100%)	3(100%)	1(100%)	1(0.79%)

Minimal change disease [19 (52.7%)] cases and FSGS [8 (22.22%)] cases were the two most common lesions presenting as nephrotic syndrome, whereas Lupus Nephritis [22 (95.65%)] cases were the predominant lesion in patients clinically diagnosed as SLE. Post-infectious GN [8 (32%)] cases and MPGN 4 (16%) were the two important lesions in nephritic syndrome.

#### Direct immunofluorescence findings

**Fig 7:**
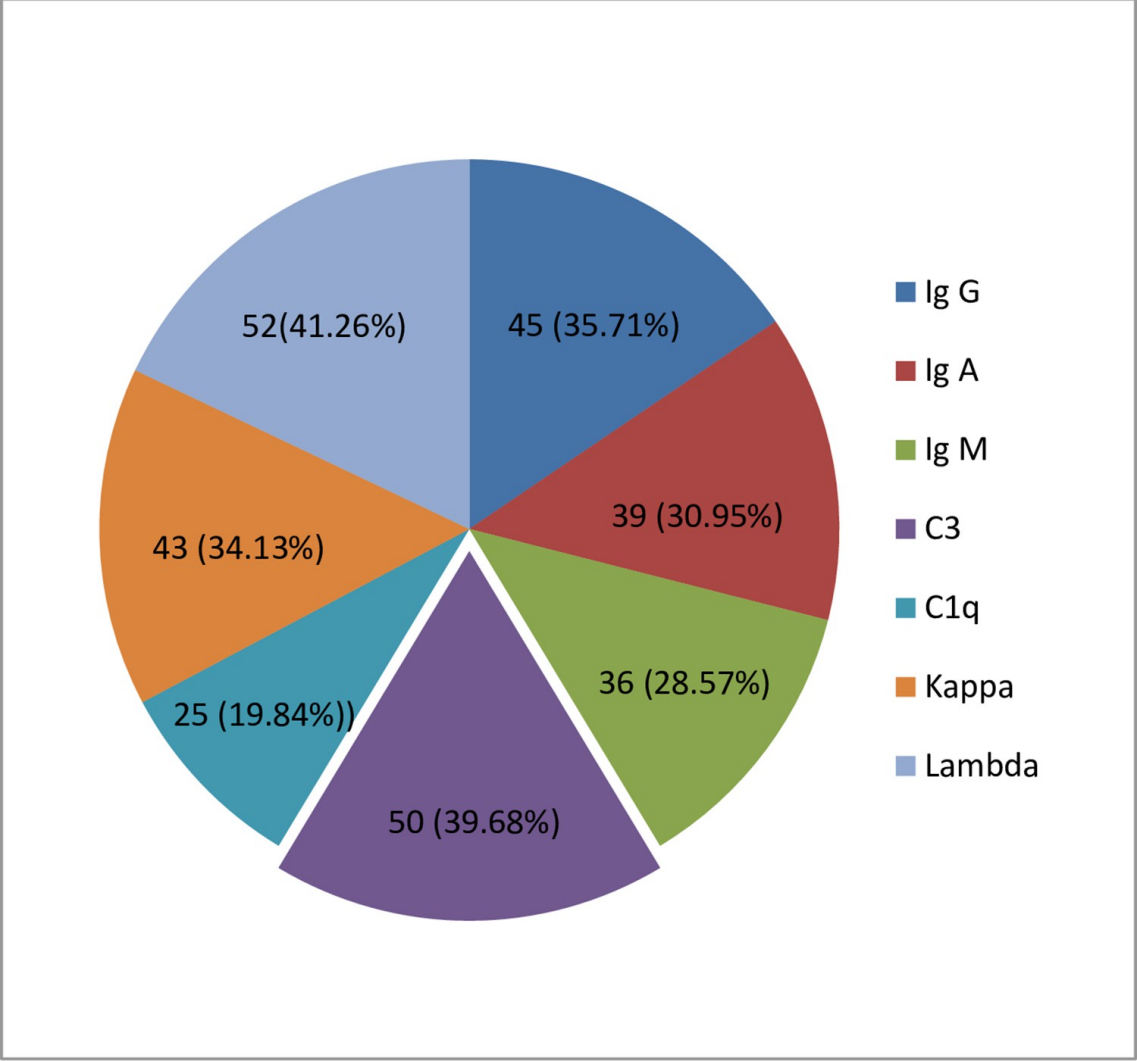
Percentage positivity of different immunoglobulins. Note: 2+ or more were considered positive on DIF.

Of the 126 cases, direct immunofluorescence microscopy was done in all cases. The immunofluorescence microscopy findings in kidney biopsies are shown in Fig 7.

The frequences of C3, Ig G, Ig A, Ig M, and C1q which were seen in positive samples, accounted for 39.68%, 35.71%, 30.95%, 28.57%, and 19.84% respectively. Of the optional markers, Lambda was positive in 41.26% and Kappa was positive in 34.13%.

**Fig 8:**
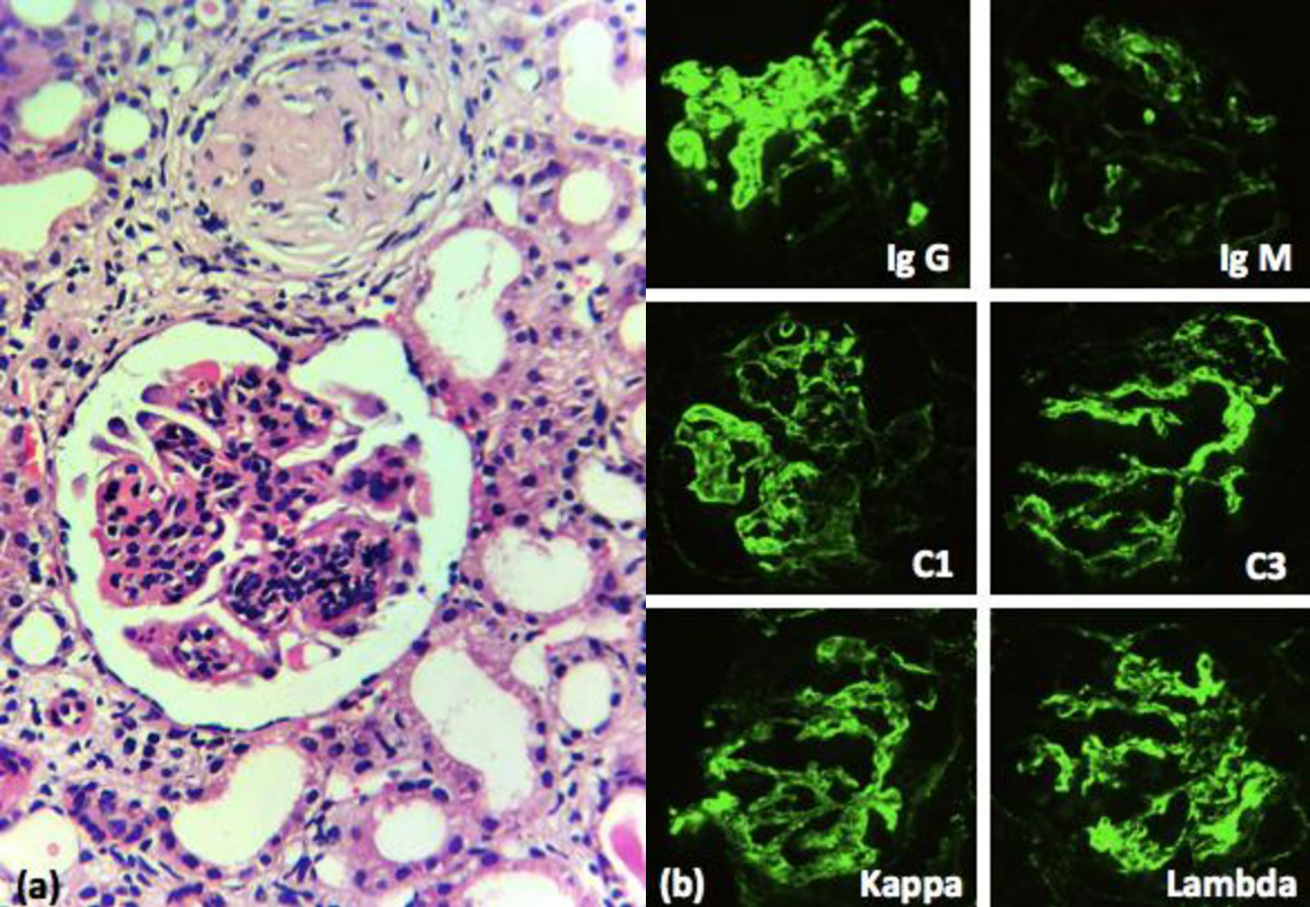
a Lupus nephritis Class IV with mesangial and endocapillary proliferation (H&E stain x400). b. DIF shows full house positivity.

**Fig 9:**
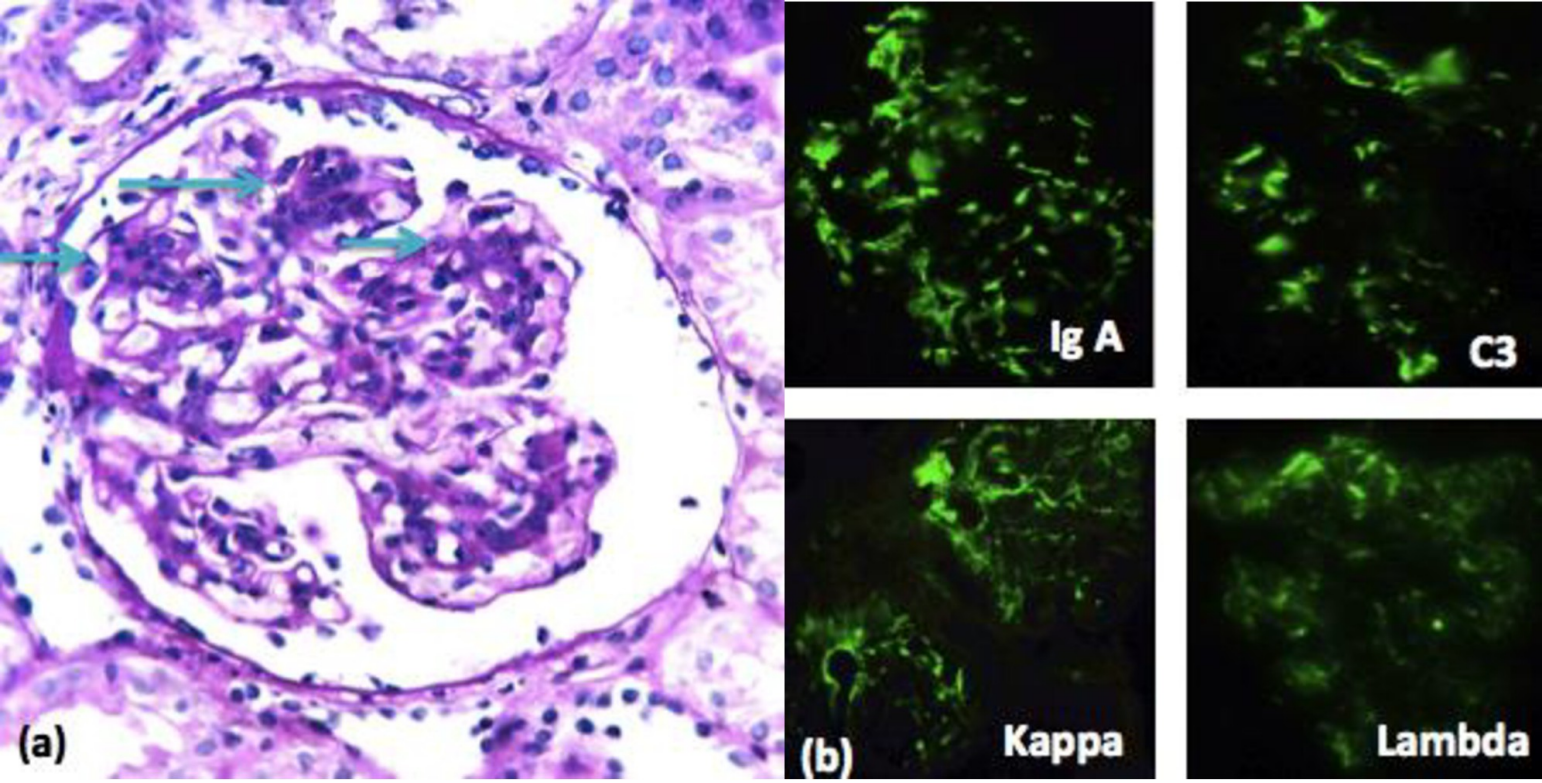
a. Ig A nephropathy with increased mesangial matrix and cellularity (arrows) (PAS stain x400). b DIF shows strong positivity with Ig A, C3, kappa and lambda.

On Direct Immunofluorescence (DIF), Lupus nephritis showed full house positivity in almost all cases with maximum positivity in Ig G (82.14%) followed by C3 (78.57%) and C1q (75%) respectively. (Fig 8) Ig A nephropathy cases showed strong positivity in Ig A (100%) and C3 (37.5%) **(Fig 9)**. In contrast, MCD cases showed negativity in all DIF parameters.

The pattern of Glomerular disease according to direct immunofluorescence microscopy is shown in Table 4.

**Table 4:** Frequency of positivity of immunoglobulins in different kidney diseases (N = 126).

FINAL DIAGNOSES	FREQUENCY	IgG (P %)	IgA (P %)	IgM (P %)	C3 (P %)	C1q (P %)	Kappa (P %)	Lambda (P %)
**Lupus Nephritis**	**28(22.22%)**	82.14%	60.71%	35.71%	78.57%	75%	75%	75%
**Minimal Change Disease**	**22(17.46%)**	0%	0%	22.73%	0%	0%	0%	4.55%
**IgA Nephropathy**	**16(12.69%)**	12.5%	100%	37.5%	37.5%	0%	43.75%	68.75%
**Focal Segmental Glomerulosclerosis**	**13(10.32%)**	7.69%	0%	38.46%	23.08%	0%	15.38%	23.08%
**Crescentic GN**	**10(7.94%)**	50%	20%	10%	30%	20%	30%	50%
**Membranoproliferative GN**	**10(7.94%)**	50%	0%	20%	70%	10%	40%	40%
**Postinfectious GN**	**10(7.94%)**	50%	10%	20%	60%	0%	10%	20%
**Membranous Nephropathy**	**3(2.38%)**	100%	0%	0%	66.67%	0%	66.67%	33.33%
**Thrombotic Microangiopathy**	**3(2.38%)**	33.33%	0%	33.33%	33.33%	33.33%	33.33%	33.33%
**Acute Kidney Injury**	**2(1.59%)**	0%	0%	0%	0%	0%	0%	0%
**Henoch-Schonlein Purpura Nephritis**	**2(1.59%)**	0%	100%	0%	0%	0%	50%	50%
**IgM Nephropathy**	**2(1.59%)**	0%	0%	100%	0%	0%	0%	50%
**Alport Syndrome**	**2(1.59%)**	0%	0%	0%	0%	0%	0%	0%
**HIV-Associated Immune Complex Kidney Disease**	**1(0.79%)**	0%	0%	100%	0%	0%	100%	100%
**Thin basement Membrane Disease**	**1(0.79%)**	0%	0%	0%	0%	0%	0%	0%
**Tubulointerstitial Nephritis**	**1(0.79%)**	0%	0%	0%	0%	0%	0%	0%
**Total**	**126(100%)**							

Note: 2+ or more were considered positive in DIF. Positive cases of MCD, FSGS, and TMA were 2+ only

## Discussion

### Demographic data and clinical presentation

Renal biopsy is a safe procedure in children and occupies a fundamental position in the management of kidney diseases [3]. In our study, there was an equal predominance of males and females. However, in other studies done by various authors, there was a slight male predominance [5, 7–11] except in a study done by Momtaz HE et al in which there was female predominance [4]. The mean age at biopsy was 12.14 ± 4.07 years, and age ranged from 1 to 18 years, which was similar to studies done in other countries [4–11]. In our study, most patients were in the age group of 11–15 years which was similar to the study done by Shrestha et al. [2].

### Clinical indications

In our study, the nephrotic syndrome was the most common indication for biopsy in children, which was similar to other research [4–11]. Similarly, in a study done by Wannous H et al, the steroid-resistant nephrotic syndrome was the most common cause of renal biopsy after treatment failure [3]. However, in other studies, steroid-sensitive cases were most commonly biopsied. [4, 7, 11].

Glomerular disease according to clinical indications showed that Minimal change disease (52.7%) and FSGS (22.22%) were the two most common diseases presenting with nephrotic syndrome whereas Lupus Nephritis (95.65%) was the predominant disease in suspected SLE. Post-infectious GN (32%) and MPGN (16%) were the two important diseases presenting with nephritic syndrome. These findings were similar to the study done by Alyousef A et al [12].

In our study, ANA/ds-DNA was done in 54 cases of which 23 (42.59%) cases were positive and all were cases of Lupus nephritis. In a similar study done by Karki et al. on 38 patients with SLE, Antinuclear antibody (ANA) was positive in all 38 (100%), Anti-dsDNA seen in 18 (47.4%) cases [13]. Pediatric renal biopsy studies showing demographic details, indications, and histopathological spectrum are illustrated in Table 5.

**Table 5:** Pediatric renal biopsy studies showing demographic details, indications, and histopathological spectrum.

Study	This Study 2021	Momtaz HE et al 2021 [4]	Arapović A et al 2020 [5]	Priyadarshini L et al 2019 [7]	Kanodai KV et al 2015 [8]	Moorani KN et al 2010 [9]	Santangelo et al 2018 [11]	Lee SA et al 2017 [10]
**Study period**	**2016–2021**	**2007–2017**	**2008–2017**	**2010–2018**	**2008–2013**	**2005–2009**	**1979–2014**	**1987–2014**
**Region**	**Nepal**	**Iran**	**Croatia**	**India**	**India**	**Pakistan**	**Italy**	**South Korea**
**Sample size (n)**	**126**	**119**	**54**	**443**	**335**	**118**	**213**	**318**
**Age (years)**	**<18**	**10.3**	**<18**	**<14**	**<14**	**<16**	**<24**	**<18**
**Male/Female**	**63/63**	**56/63**	**29/25**	**279/164**	**228/107**	**62/56**	**121/92**	**205/113**
**Most Common Indication**	**Nephrotic syndrome**	**Nephrotic syndrome**	**Nephrotic syndrome**	**Nephrotic syndrome**	**Nephrotic syndrome**	**Nephrotic syndrome**	**Nephrotic syndrome**	**Recurrent hematuria**
**Histopathology**								
**Primary GN**	**68.25**	**93.3**	**67**	**88.85**	**81.79**	**84.74**	**70.6**	**73.88**
**FSGS**	10.31	**32.7**	11.1	22.12	8.02	**29.66**	11.6	3.4
**MCD**	**17.46**	16.8	16.7	**47.63**	6.93	32.2	18.1	17.92
**MN**	2.38	5.1	3.7	1.58	3.28	8.47	3.3	1.2
**PIGN**	7.94	3.4	-	0.68	9.6	3.38	7.9	2.5
**Crescentic GN**	7.94	5.1	1.9	-	10.94	-	3.7	-
**MPGN**	7.94	13.4	1.9	1.35	13.5	7.16	**20.9**	10.3
**IgAN**	12.69	10.9	**24.1**	4.96	6.2			**27.9**
**IgMN**	1.58	-	**-**	4.06	14.96	2.54	3.2	3.46
**DPGN**	**-**			1.12				
**MesPGN**	**-**	-	1.9	1.52	**24.5**	1.33		7.2
**RPGN**				2.25			1.9	
**Congenital NS-Finnish type**	**-**	5.9	**-**	**-**	**-**	**-**	**-**	**-**
**C1q N**	**-**		1.9	1.58	**-**	**-**	**-**	**-**
**C3 G**	**-**	-	1.9		**-**	**-**	**-**	**-**
**FSNGN**	**-**		1.9		**-**		**-**	**-**
**Secondary GN**	**29.36**	**6.7**	**20.4**	**9.69**	**16.12**	**14.36**	**16.7**	**15**
**LN**	**22.22**	**6.7**	1.9	**5.19**	**7.76**	**9.32**	5.1	1.5
**Thrombotic Microangiopathy**	2.38	**-**	-	0.68	-	-	1.4	-
**HSPN**	1.59	**-**	**14.8**	1.12		2.52	6.5	**12.2**
**AKI**	1.59	**-**	**-**	-	-	-	-	-
**HIV associated ICKD**	0.79	**-**	**-**	-	-	-	-	-
**Amyloidosis**	-	**-**	**-**	-	0.89	-	-	-
**TIN**	0.79	**-**	3.7	2.25	-	-	3.7	1
**ATN**	-	**-**	**-**	0.45	-	--	-	0.3
**HUS**	-	**-**		-	6.26	2.52	-	-
**HTN**	-	**-**	**-**	-	1.19		-	-
**Miscellaneous**	**2.39**	**-**	**13**	**1.46**	**2.09**	**0.9**	**12.7**	**11.12**

FSGS (Focal Segmental Glomerulosclerosis), MCD (Minimal Change Disease), MN (Membranous Nephropathy), PIGN (Post Infectious Glomerulonephritis), Crescentic GN (Crescentic Glomerulonephritis), MPGN(Membranoproliferative Glomerulonephritis), Ig AN (Immunoglobulin A Nephropathy), Ig MN (Immunoglobulin M Nephropathy) DPGN (Diffuse Proliferative Glomerulonephritis) MesPGN (Mesangioproliferative Glomerulonephritis), RPGN (Rapidly Progressive Glomerulonephritis), Congenital NS-Finnish type (Congenital Nephrotic Syndrome Finnish Type), C1q N (Complement 1q Nephropathy), C3 G(Complement 3 Glomerulopathy), FSNGN(Focal segmental necrotizing glomerulonephritis), Secondary GN (Secondary Glomerulonephritis), LN (Lupus Nephritis), Thrombotic Microangiopathy, HSPN(Henoch-Schönlein purpura glomerulonephritis), AKI(Acute Kidney Injury), HIV associated ICKD(HIV associated Immune Complex Kidney Disease), TIN (Tubulointerstitial nephritis) ATN (Acute Tubular Necrosis) HUS (Hemolytic Uremic Syndrome) HTN (Hypertensive Nephropathy)

### Histopathological diagnoses

In this study, primary glomerulonephritides were more common than secondary glomerulonephritis, which was similar to various other studies [4, 5, 7–11, 14].

SLE was the most commonly reported pathologic diagnosis in secondary glomerulonephritis with a frequency of 22.22%. A higher frequency of childhood-onset SLE has been reported in Asians in various studies [15, 16]. In our study, it was more common in girls in an age group of 11–15 years. All of them were dsDNA/ANA positive.

MCD was the most common histopathological diagnosis in primary glomerulonephritis with a prevalence of 17.46%. It was more common among boys. They were all prevalent in the age group 11–15 years of age group. Most of them presented with nephrotic syndrome. MCD was one of the major causes of primary glomerular disease in children in Asian populations [17]. Similarly, a study was done by Priyadarshini et al [7] and Alyousef et al. [12] showed MCD as the commonest pathological diagnosis among children accounting for 47.63% and 35.5% respectively. The incidence of MCD cases in our study may have been underestimated as many patients responded to steroid treatment and were not biopsied. Most of our cases were steroid-resistant during the time of biopsy.

IgA nephropathy was the second most common diagnosis in primary glomerulonephritis with a prevalence of 12.69%. It was more common among boys in an age group of 11 to 15 years. Clinically, patients presented with nephrotic as well as nephritic syndrome, non-nephrotic proteinuria, or isolated hematuria. Similarly, other studies showed that IgA nephropathy was more prevalent among children in Asian populations [18–20]. Studies done by Arapovic et al [5] in Croatia and Lee et al. [10] in South Korea also showed IgA nephropathy as the most common diagnosis in histopathology in pediatric renal biopsies with 24.1% and 27.9% respectively.

FSGS was the third most common histopathological diagnosis in our study with a prevalence of 10.31% more commonly seen in males and age group of 11–15 years. Clinically, they presented more with features of nephrotic syndrome. Similarly, in studies done by Momtaz et al [4] and Moorani et al. [9], FSGS was the most common diagnosis with 32.7% and 29.66% respectively.

### Histopathological lesions in lupus nephritis

The role of the International Society of Nephrology and the Renal Pathology Society (ISN/RPS) 2016 classification of Lupus nephritis is to bring standardization and uniformity in the reporting of all renal pathology reports of Lupus nephritis, better clinical-pathological correlation, and its therapeutic and prognostic implications [21, 22].

According to the International Society of Nephrology and the Renal Pathology Society (ISN/RPS) 2016 classification of Lupus nephritis in our study, Class IV (57.1%), followed by Class III (35.7%) were most common. Activity index <12 in 78.6% and chronicity index <4 in 92.8% were primarily observed in our study. In various studies, the most common histology of lupus nephritis in Asian children was Class IV lupus nephritis (39.4–54%), which is also true in other Western countries such as the United States (38%) and Canada (46%) [23]. Class IV Lupus nephritis is the most common Lupus nephritis in South East Asian countries as shown in Table 6 [2, 9, 12, 23–29].

**Table 6:** Histopathological classification of childhood-onset lupus nephritis in Southeast Asia.

ISN/RPS 2016 Classification Of Lupus Nephritis	This study 2021	Tan et al 2021 [25]	Lim et al 2020 [26]	Shrestha D et al 2018 [2]	Mutalik PP et al 2015 [24]	Dung et al 2012 [29]	Vachvanichsanong et al 2011 [27]	Gulay and Dans et al 2011 [28]	Moorani KN et al 2010 [9]
**Country**	**Nepal**	**Singapore**	**Malaysia**	**Nepal**	**India**	**Vietnam**	**Thailand**	**Philippines**	**Pakistan**
**Number of cases (n)**	**28**	**21**	**44**	**10**	**11**	**29**	**87**	**24**	**11**
**CLASS I**	-	-	2.3	-	-	-	5.7	-	9
**CLASS II**	3.6	-	18.2	-	-	-	21.8	25	9
**CLASS III**	35.7	23.8	25	30	27.27	24.2	8	20.8	**27**
**CLASS IV**	**57.1**	33	**36.4**	**70**	**63.63**	**60**	**56.3**	**50**	**27**
**CLASS V**	3.6	4.8	4.5	-	9.09	-	8	4.1	**27**
**CLASS VI**	-			-	-	3			-
**Mixed III/IV + V**		**34.8**	13.7				-	-	

### Classification of crescentic glomerulonephritis

The histopathologic classification of Crescentic glomerulonephritis is useful for prognosis [30, 31]. In our study, out of 10 cases of crescentic glomerulonephritis, the highest frequencies were of Type III (Pauci immune) (50%), followed by Type I (Anti-GBM) (30%) and Type II (Immune complex) (20%). Generally, pauci-immune glomerulonephritis is the most common crescentic glomerulonephritis in all age groups, and a majority of them are ANCA positive [32]. However, in a study done by Ulrike Mayer et al., out of 60 crescentic glomerulonephritis, Type II (Immune complex) (75%) was most common, followed by Type III (Pauci immune) (16.67%), Type I (Anti-GBM) (1.67%) and Undetermined (6.67%) [33].

### Histopathological findings in IgA nephropathy

The updated Oxford Classification (MEST-C) 2016 has therapeutic and prognostic implications [34, 35]. According to the updated Oxford Classification (MEST-C) 2016, in our study, the most common lesions were M1, EӨ, SӨ, TӨ, and CӨ. However, in a study done by Moriyama [34] et al., MӨ, and S1 were more common than M1 and SӨ, but EӨ, TӨ, and CӨ were more in their study, which was similar to our research.

### Direct immunofluorescence findings

Light microscopy gives the morphological pattern only, so to be more precise, we need to correlate light microscopy findings with clinical features; immunofluorescence and electron microscopy [36]. Direct immunofluorescence microscopy was done in all renal biopsies. The high frequency of C3 (39.68%), Ig G (35.71%), Ig A (30.95%), Ig M (28.57%), and C1q (19.84%) were seen in positive samples. Of the optional markers, Lambda was positive in 41.26% and Kappa was positive in 34.13%. Similarly, the study done by Momtaz et al and Zafar et al. showed IgG positivity followed by C3 positivity with the highest frequency in most of the DIF positive cases as shown in Table 7 [4, 37].

**Table 7:** DIF Frequency in various studies.

	DIF Frequency In Various Studies		
DIF	This study 2021	Momtaz HE et al 2021 [4]	Zafar F et al 2021 [37]
Country	Nepal	Iran	Pakistan
**Number of cases (n)**	**126**	**87**	**387**
**IgG**	**35.71%**	**29.41**	**9.3**
**IgA**	30.95%	10.92	2.3
**IgM**	28.57%	21.01	14
**C3**	**39.68%**	26.89	6.2
**C4**	-	6.72	-
**C1q**	19.84%	5.04	0.8
**Kappa**	34.13%	-	-
**Lambda**	41.26%	-	-

On DIF, Lupus nephritis showed full house positive in almost all cases with maximum positivity in Ig G (82.14%) followed by C3 (78.57%) and C1q (75%) respectively. Ig A nephropathy showed strong positivity to Ig A (100%) and C3 (37.5%). In contrast, MCD showed negativity in all DIF parameters. Moreover, a study done by Buch CA et al. also showed full house positivity in Lupus nephritis, negative DIF in all cases of MCD except 2 cases, and strong Ig A positivity in Ig A nephropathy [36]. Similarly, a study done by Zafar et al also showed MCD with full house negativity in DIF and Lupus nephritis showed maximum positivity in C3 [37].

## Conclusion

- The most common indication of pediatric renal biopsy was nephrotic syndrome.
- MCD was the most frequent diagnosis among Primary GN.
- Lupus nephritis was the most common overall diagnosis and Secondary GN.
- Ig A nephropathy was the second most common diagnosis in Primary GN.
- Lupus nephritis showed full house positivity, minimal change disease showed full house negativity in all DIF parameters, whereas immunoglobulin A nephropathy showed 100% positivity in Ig A in DIF.
- Clinical data, light microscopy, and DIF play an integral role in the overall final diagnosis.
- **The data of this study is based on a single center experience and do not represent prevalence of kidney disease in children in our country.**

### Limitations of the study

- The unavailability of electron microscopy for the examination of equivocal cases is a challenge to a developing country like Nepal.
- Most of the patients in our case were from rural areas and presented to the hospital at the late presentation of the disease in severe form with full-blown features.
- Minimal change disease cases were already given steroid treatment before biopsy. Many of them were steroid-resistant during the time of biopsy. Therefore the incidence of MCD may be underestimated.

## Supporting information

S1 File (XLSX)

## List of abbreviations

AKI: Acute kidney injury
ANA: Antinuclear antibody
ANCA: Antineutrophil Cytoplasmic Antibodies
Anti-dsDNA: Anti-double-stranded deoxyribonucleic acid
Anti-GBM: Anti- Glomerular Basement Membrane
AS: Alport syndrome
ATN: Acute tubular necrosis
C1q: Complement component 1q
C3: Complement component 3
C4: Complement component 4
Crescentic GN: crescentic glomerulonephritis
DIF: Direct immunofluorescence
DPGN: Diffuse proliferative glomerulonephritis
EM: Electron-microscopy
FFPE: Formalin-fixed and paraffin-embedded
FITC: Fluorescein isothiocyanate
FRNS: Frequently relapsing nephrotic syndrome
FSNGN: Focal segmental necrotizing glomerulonephritis
FSGS: Focal segmental glomerulosclerosis
GN: Glomerulonephritis
HE: Hematoxylin and Eosin
HIV: Human Immunodeficiency Virus
HSPN: Henoch-Schönlein purpura glomerulonephritis
HUS: Hemolytic–uremic syndrome
HTN: Hypertensive nephropathy
ICKD: Immune Complex Kidney Disease
IF: Immunofluorescence
IgA: Immunoglobulin A
IgG: Immunoglobulin G
IgM: Immunoglobulin M
ISN/RPS: International Society of Nephrology and the Renal Pathology Society
LM: Light-microscopy
LN: Lupus nephritis
MCD: Minimal change disease
MesPGN: Mesangioproliferative glomerulonephritis
MEST-C: Mesangial cellularity, Endocapillary proliferation, Segmental Sclerosis, Tubular atrophy, Crescent
MGN: Membranous glomerulopathy
MPGN: Membranoproliferative glomerulonephritis
NS: Nephrotic syndrome
PAS: Periodic acid- Schiff
PBS: Phosphate-buffered saline
PIGN: Post-infectious glomerulonephritis
RPGN: Rapidly progressive glomerulonephritis
SDNS: Steroid-dependent nephrotic syndrome
SLE: Systemic Lupus Erythematosus
SPSS: Statistical Package for the Social Sciences
SRNS: Steroid-resistant nephrotic syndrome
TBMN: Thin basement membrane nephropathy
TIN: Tubulointerstitial nephritis
WHO: World Health Organization
